# Epidemiology of Congenital Cholesteatoma: Surveys of the Last 17 Years in Japan

**DOI:** 10.3390/jcm13051276

**Published:** 2024-02-23

**Authors:** Yoshinori Kadowaki, Shinsuke Ide, Takeshi Nakamura, Takumi Okuda, Hideto Shigemi, Takashi Hirano, Kuniyuki Takahashi, Masashi Suzuki

**Affiliations:** 1 Department of Otolaryngology-Head and Neck Surgery, Faculty of Medicine, Oita University, Oita 879-5593, Japan; dowaki615@oita-u.ac.jp (Y.K.); hideto-shigemi@oita-u.ac.jp (H.S.); suzukim@oita-u.ac.jp (M.S.); 2 Department of Otolaryngology-Head and Neck Surgery, Faculty of Medicine, Miyazaki University, Miyazaki 889-1601, Japan; shinsuke_ide@med.miyazaki-u.ac.jp (S.I.); takeshi_nakamura@med.miyazaki-u.ac.jp (T.N.); kuniyuki_takahashi@med.miyazaki-u.ac.jp (K.T.); 3 Department of Otolaryngology-Head and Neck Surgery, Miyazaki Prefectural Miyazaki Hospital, Miyazaki 880-0017, Japan; takumi_okuda@med.miyazaki-u.ac.jp

**Keywords:** congenital cholesteatoma, epidemiology, incidence, demographics, staging

## Abstract

(1) **Background**: The incidence of congenital cholesteatoma (CC) has rarely been discussed, particularly from a demographic viewpoint. Therefore, we conducted an epidemiological study of CC using local medical characteristics. (2) **Methods**: The participants were 100 patients (101 ears) who underwent initial surgical treatment at university hospitals in two rural prefectures between 2006 and 2022. A total of 68% of the patients were males and 32% were females, with a median age of 5 years. We reviewed the medical records for the date of birth, date of surgery, stage of disease, and first symptoms of the disease. (3) **Results**: The total incidence of CC was calculated to be 26.44 per 100,000 births and tended to increase. No significant difference was found between the incidences in the two prefectures. The number of surgeries performed was higher in the second half of the study period. No difference in the stage of progress was observed based on age. (4) **Conclusions**: The incidence of CC was estimated to be 26.44 per 100,000 newborn births. The number of patients with CC tended to increase; however, this can be attributed to an increase in the detection rate rather than the incidence.

## 1. Introduction

Congenital cholesteatoma (CC) is a white retrotympanic mass associated with a normal tympanic membrane without a history of otorrhea, perforation, or otologic surgery [1]. In 1953, CC of the middle ear was first described by House [2]. In 1965, Derlacki and Clemis [3] reported ten cases of CC and listed “no prior history of tympanic membrane perforation” and “no prior history of infection” as strict conditions among the diagnostic criteria for diagnosing CC and differentiating it from acquired lesions. However, in 1986, Levenson et al. modified the criteria so that a history of otitis media did not exclude the diagnosis of CC [4]. In addition to these diagnostic criteria, in 2002, Potsic et al. proposed a classification of CC [5] that is now commonly used. More recently, the European Academy of Otology and Neurootology/Japan Otological Society (EAONO/JOS) provided joint consensus statements on the definitions, classification, and staging of middle ear cholesteatoma, including CC [6]. The incidence of various congenital diseases has been demonstrated in epidemiological studies; nevertheless, the incidence of CC has rarely been discussed, even though it is a congenital disease. Therefore, this study was aimed at investigating the epidemiology of CC in Japan based on demographics.

## 2. Materials and Methods

This study was approved by the ethics committees of Oita University and Miyazaki University (approval number: 2487).

A total of 101 ears of 100 patients who underwent initial surgical treatment for CC at the Oita and Miyazaki University Hospitals between January 2006 and December 2022 were recruited for this retrospective study. The participants were limited to those born in the respective prefecture. We reviewed the medical records of the patients for age, sex, laterality, date of birth, date of surgery, disease stage, operation records, and first symptoms of the disease. The disease was diagnosed based on the 2015 JOS classification criteria: cholesteatoma that develops behind an intact tympanic membrane; a history of otitis media does not necessarily exclude CC, but cases with previous otologic procedures should be excluded [7]. The hearing level was calculated using the air conduction thresholds of the 4-frequency average: 0.5, 1, 2, and 3 kHz. We do not usually measure the 3 kHz hearing level in audiometry in Japan; therefore, we used the average values of 2 kHz and 4 kHz as the 3 kHz hearing level.

The participants were analyzed regarding the following items:

1. *Incidence of CC per newborn births*: The number of newborn births per year in Oita and Miyazaki prefectures was obtained from the data available on their websites (https://www.pref.oita.jp/uploaded/attachment/2008926.pdf, accessed on 22 February 2024; https://statdb.me/births/45000/, accessed on 29 January 2024). In this incidence analysis, the lag between birth and surgery may have compromised the accuracy of the results. Therefore, we calculated the incidence in patients born between 2001 and 2017, i.e., 5 years backward with the median age at surgery during this research period. Patients were divided by year of birth from 2001 to 2017, and the incidence per 100,000 births was calculated for each year. The figures for each prefecture and the total incidence rates for both prefectures were also calculated. In addition, the transition of the incidence was analyzed by measuring the correlation coefficients between the number of patients and the year of birth.
2. *Number of surgeries by year*: The number of surgeries performed between 2006 and 2022 was calculated, and the patients’ ages at the time of surgery were sorted. Subsequently, changes in the number of surgeries and age were measured at the time of surgery.
3. *First opportunity for medical examination*: Most patients were suspected of having CC when they initially visited a nearby clinic and were referred to the respective university hospital. Opportunities for medical examinations led to the diagnosis of the disease and were divided into the following four categories:
  - Otitis media: the symptoms of otitis media, including otalgia and otorrhea.
  - Routine examination: a white mass was found on the tympanic membrane by routine examinations when the patient was examined for symptoms other than those of the ear.
  - Hearing loss: patients who became aware of hearing impairment or whose hearing loss was noted during a hearing checkup.
  - Others.

Four patients with unknown backgrounds were excluded. In addition, the age at that time was also divided into four categories: 0–2 years (before entering kindergarten), 3–5 years (kindergarten), 6–9 years (lower grades of elementary school), and 10 years and above (upper grades of elementary school and above).

### Disease Progression

Disease staging was classified according to the JOS classification criteria [7]: stage Ia, cholesteatoma confined to the anterior half of the tympanic cavity; stage Ib, cholesteatoma confined to the posterior half of the tympanic cavity; stage Ic, cholesteatoma involving both sides of the tympanic cavity; stage II, cholesteatoma involving two or more sites; stage III, cholesteatoma with intratemporal complications and pathologic conditions; and stage IV, cholesteatoma with intracranial complications. One patient with unknown progression was excluded from the analysis. One ear with an unknown extent of the lesion was excluded from the study. We also examined the correlation between age at surgery and disease progression.

Statistical analyses were performed using EZR ver. 1.61 (Saitama Medical Center, Jichi Medical University, Saitama, Japan). Statistical significance was set at *p* < 0.05.

## 3. Results

### 3.1. General Aspects

This study comprised 69 males (68%) and 31 females (32%). Regarding the affected side, 51 ears were in the right ear and 50 ears were in the left. The mean age of the patients at surgery was 6.7 ± 4.6 years, and the median age was 5 years. One case (1%) had a bilateral lesion. Fifty-three percent of the patients had an assessment of their preoperative hearing level using pure-tone audiometry. The mean value was 48.2 dB hearing level, and all cases with hearing loss had conductive hearing loss.

### 3.2. Incidence of the CC per Newborn Births

**Figure 1:**
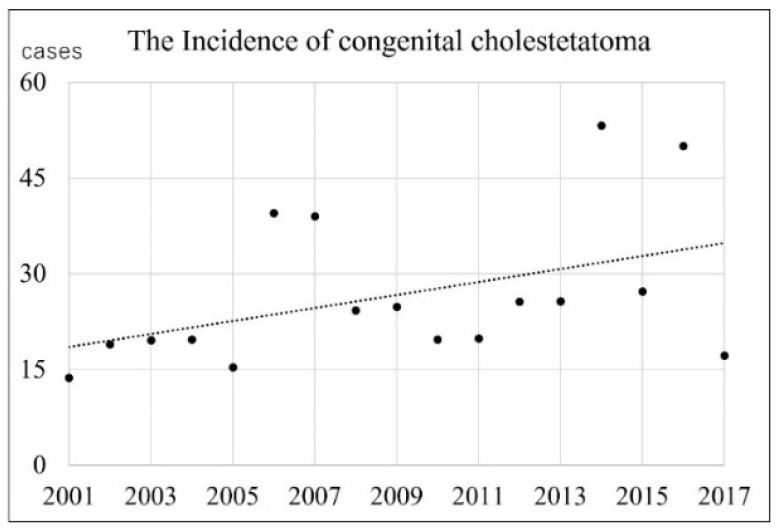
Transition in the incidence of CC. Each point represents the incidence of patients born in that year. These values are significantly positively correlated (R = 0.53, *p* = 0.03), indicating a trend in the incidence over this period.

Table 1 shows the number of newborn births, the number of patients with CC, and the incidence rate per 100,000 newborn births each year. The number of newborn births in both the Oita and Miyazaki prefectures has been declining almost every year, with a 20% decrease from 2001 to 2017. A total of 47 patients with CC were born in the Oita prefecture and 42 in the Miyazaki prefecture during this period. The mean incidence rate per 100,000 newborn births was 28.09 and 24.80 in the Oita and Miyazaki prefectures, respectively. Consequently, the total disease incidence rates in both prefectures during the study period were calculated to be 26.44. There was no statistically significant difference in the incidence rates between the two prefectures (*t* test, *p* = 0.414). There was a significant positive correlation between the year of birth and the total disease incidence. Spearman’s rank correlation coefficient was 0.53 (*p* = 0.03) (Figure 1). Therefore, its incidence increased from 2001 to 2017.

### 3.3. Number of Surgeries by Year

**Figure 2:**
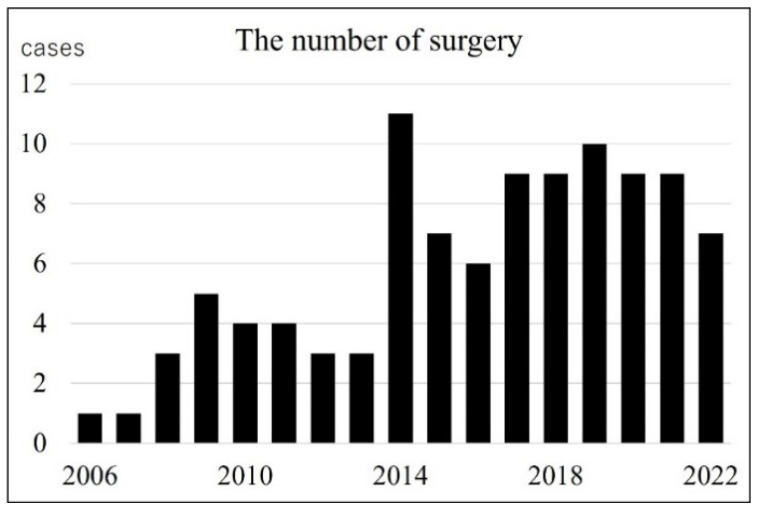
Number of surgeries by year. The number of surgeries increased significantly in the latter half of the period, starting in 2014.

**Figure 3:**
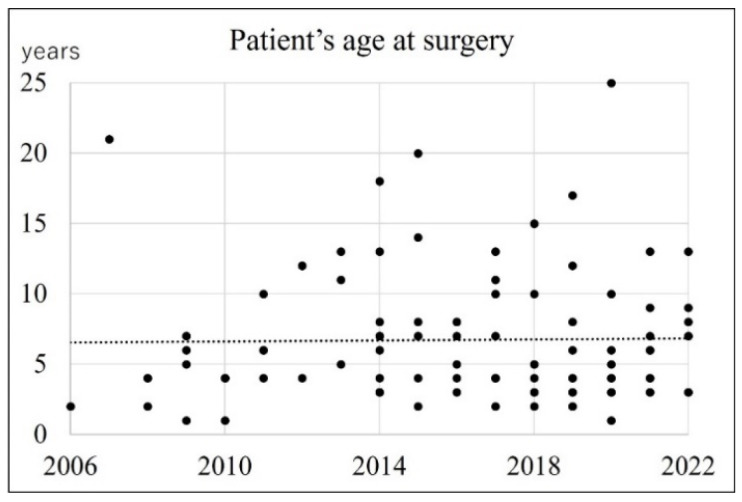
Patients’ ages at surgery. Each point represents the age of patients who underwent surgery in that year. There was no correlation between the two items. (R > −0.1, *p* = 0.099).

During the study period, the total number of surgeries performed at the two facilities ranged from 1 to 11 per year (Figure 2). Comparing before and after 2014, the number of surgeries performed in each year was significantly higher in the latter half (Mann–Whitney u test, *p* < 0.001). Patients’ ages at surgery ranged from 1 to 25 years. Figure 3 shows the patients’ ages divided by the year of surgery during the study period. There was no significant correlation between the year of surgery and the mean age of patients (Spearman’s rank correlation coefficient > −0.1, *p* = 0.099). Five of the relatively older patients (12–25 years) had ossicular malformations. Ossicular malformations were defined according to the definition of Kojima et al.: absence or deformity of the ossicles not resulting from bone destruction by the cholesteatoma [8]. Referring to operation records, cases in which the ossicular defect was distant from the cholesteatoma were selected for ossicular malformations. The mean age at the time of surgery of cases with ossicular malformations was significantly higher (17.8 years) than that in those without (6.1 years) (Mann–Whitney u test, *p* < 0.01). However, in this study, it was not impossible to analyze open-type or closed-type cases because many cases were not clearly recorded in the operation records.

### 3.4. First Opportunity for Medical Examination (Figure 4)

Of the 97 ears, 25 (25.8%) had otitis media, 32 (33.0%) underwent routine examination, 37 (38.1%) had hearing loss, and 3 (3.1%) had other reasons as the first opportunity for the diagnosis of CC. Before entering elementary school, otitis media and routine examination accounted for most cases (92.3%); however, after entering elementary school, most cases were due to hearing loss (75.6%). In addition, the proportion of cases due to hearing loss increased further with age (89.5%). The other three cases were found incidentally. One case was detected with a white mass on the tympanic membrane during a school medical checkup, and two cases were found using computed tomography (CT) images: one had a head CT taken when he was involved in a traffic accident, and the other had bilateral lesions identified on the opposite side on a temporal bone CT.

**Figure 4:**
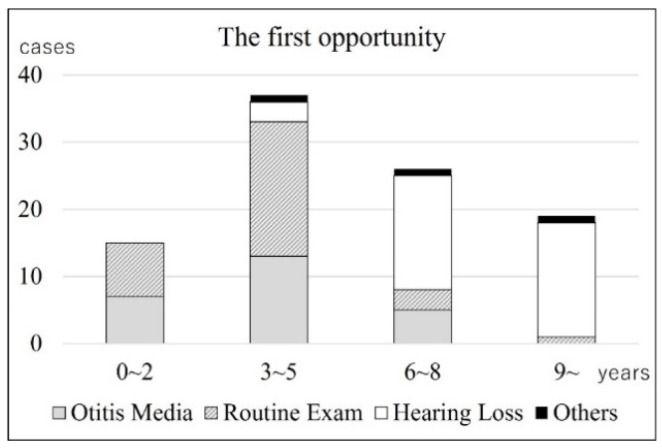
First opportunity for medical examination. In the age group under 5 years, otitis media and routine examination were the most common reasons for diagnosis, but hearing loss was the most common in the age group of 6 years and above.

### 3.5. Disease Progression (Figure 5)

A total of 47 ears were classified as stage I (Ia: 18 ears; Ib: 21 ears; Ic: 8 ears) and 53 ears as stage II. Among the stage II cases, 51 ears (96%) had attic involvement and 23 ears (43%) had mastoid involvement. No cases were classified as stage III (inner ear or facial nerve complications) or IV (intracranial complications). The average age by stage was 6.8 years for stage|and 6.6 years for stage II, with no significant difference between the two groups (Mann–Whitney u test, *p* = 0.39).

**Figure 5:**
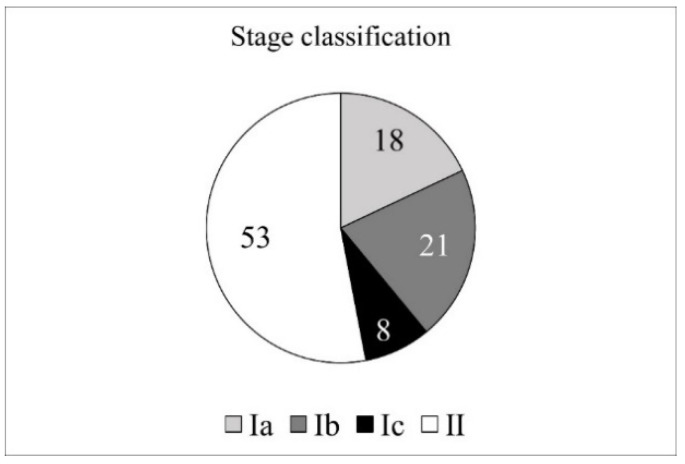
Stage classification. Stage classification by the JOS staging system. There were no stage III and IV cases.

## 4. Discussion

There are various theories regarding the etiology of CC, and Koltai et al. divided these into four main theories: implantation, invagination, metaplasia, and epidermoid formation [9]. Of these, the epidermoid formation theory described by Michaels [10] is the most accepted today [11]. However, no consensus has yet been established about the origin of CC [11,12]. As mentioned above, CC is considered a pathological condition different from acquired cholesteatoma and is relatively rare [12]. Nevertheless, the incidence of CC has only been discussed in comparison to that of acquired cholesteatoma thus far. Komori [13] reported that CC accounted for 13% of all cases of cholesteatoma, including adult cases, in a Japanese nationwide study, and Kazahaya [14] reported that CC was 2–5% of all cholesteatoma cases. Denoyelle [1] reported that the proportion of CC in pediatric cholesteatoma is 1–5%. However, these numbers cannot be used directly to estimate the incidence of CC, as there seems to be a difference in the rate of surgical treatment between congenital and acquired cases. That is, in cases of suspected CC, the common practice is to consider surgical treatment and refer the patient to major hospitals. However, this is not necessarily the case with acquired cholesteatoma. Previous reports also mentioned that it was difficult to determine the actual incidence of CC [12,14,15]. As mentioned above, the reason for the difficulty in discussing the incidence of CC is that it is impossible to examine the tympanic membrane of all newborns, and there are no other screening methods, such as blood tests or hearing screening. Only Tos [16] reported the incidence of CC using demographic statistics. As there were 13 cases over 30 years of age in the medical field of 350,000 people, he calculated the annual incidence rate to be one case per one million people. The Oita and Miyazaki prefectures are neighbors located in the eastern area of Kyushu Island, Japan. Each prefecture contains 1% of the national population and can be depicted as a “rural area” in Japan. In addition to geographical proximity, both prefectures are rural areas with little population movement and have a medical system in which all patients with CC are concentrated at the only university hospital in the prefecture. We conducted this study focusing on the fact that this “closed” environment is even more advantageous for epidemiological investigations of CC.

Regarding the participant’s demographics, 68% were males and 32% were females. The male-to-female ratio was 2:1 in both Japanese nationwide research [13,17] and in a systematic review of the English literature [18], similar to the ratio in our study, with a predominance of males. The reason for this predominance has been unclear, and none of the four theories listed at the beginning of this section can explain it [11]. The average age of the patients at surgery (6.7 years) was almost the same as that in the systematic review [18] (6.6 years). The average age in the nationwide research [17] was higher at 8.2 years. However, the median age was 6.0 years, suggesting that some adult patients included in the study may have elevated the average age. A bilateral lesion was observed in 1% of all cases. Out of 1469 cases in the Gilberto report [18], 28 (2%) were found to have bilateral lesions; the Distinguin report [11] identified 2 out of 171 cases (1%); and the Nelson report [19] reported 2 out of 119 cases (1.7%), which indicated similar proportions.

The incidence of CC was calculated to be 26.44 cases per 100,000 newborn births. No significant difference existed between the incidences in each prefecture (Oita: 28.09; Miyazaki: 24.80), ensuring the reliability of these values. There was a significant positive correlation between the year of birth and incidence from 2001 to 2017, indicating that the incidence has been increasing. However, it is unlikely that the incidence of congenital diseases will increase over such a short period, and it is more likely that there is an increase in the detection rate than in the incidence. As Im et al. [20] and Morita et al. [21] mentioned, the development of the ENT endoscopic system and expanding recognition of the disease may have contributed to the elevation of the detection rate. Hence, it is believed that this incidence will reach its upper limit before long or that it has already been reached.

According to the calculation method of Tos [16], the combined population of both prefectures was 2.3 million people, and there were 89 patients in the 17 years of the target period, with an annual incidence of 2.3 cases per million of the population. Ethnic differences in the prevalence of acquired cholesteatoma have been reported: highest in Caucasians, followed by Africans, and very low in Asians [12]. Although it is uncertain whether there are differences in the incidence of CC among ethnic groups, this number was approximately twice that reported by Tos. However, his research period was 30 years, starting in 1965, so there may not be a big difference between each result, considering the recent improvement in the detection rate.

Accompanied by an increasing incidence, the number of surgeries performed increased significantly in the latter half of 2014. Denoyelle et al. [1] also reported that the incidence of CC increases with more effective screening. Similar to the incidence, surgery is likely to reach an upper limit. Some reports have pointed out that the age at disease detection is decreasing [20,21]; however, there was no significant change in the age at detection during our study period. Patients with ossicular malformations (5 of the 101 ears [5%]) were significantly older at the time of surgery than those without. Kojima et al. reported that ossicular malformations were found in 5 (8.8%) of 57 ears, and many were associated with open-type cholesteatoma. Furthermore, they considered that open-type CC, unless it develops inflammation, can remain asymptomatic for a long time without compressing or destroying surrounding structures because debris is removed through the eustachian tube by the self-cleaning mechanism of the tympanic cavity [8]. Teranishi et al. reported a case of CC that remained unchanged in size for 12 years. However, in cases of a deficiency of the ossicular chain, CC was the closed type. Consequently, they considered that the ossicular chain discontinuity was caused by erosion associated with CC rather than ossicular anomalies [22]. As mentioned above, it is not easy to completely distinguish between ossicular anomalies and lysis [11].

The first opportunity for a medical examination varies with age. Particularly, the proportion of patients with “hearing loss” significantly increased after the patients entered elementary school. There may be individual factors, such as the patients becoming less susceptible to otitis media or becoming more aware of their own hearing loss as they grow up, and the disease factor, which is the progression of hearing loss accompanied by cholesteatoma growth. Morita et al. [21] reported that 52% of patients had a lesion during routine examinations of their ears, and 42% were informed of hearing loss during their medical examinations. Gilberto et al. [18] reported that 34% of patients were asymptomatic, 26% had hearing loss, and 23% had a history of otitis media with effusion or acute otitis media. As mentioned above, in addition to individual and disease factors, school screening can be a major factor in disease detection, and its importance has been emphasized.

According to the JOS classification criteria in this staging study, 47% were stage I, 53% were stage II, and there were no cases of stage III or IV neurological or intracranial complications. This is almost similar to that reported by Morita et al. [17] (stage I, 46%; stage II, 52%; and stage III, 2%), and the complication rate is lower than that of acquired cholesteatoma. Some authors [14,18,23] reported that cholesteatoma size increased and staging progressed with patients’ ages according to the Potsic classification. However, no relationship between disease progression and the patients’ ages was observed in this study. This may be due to the difference between the JOS and Potsic classifications. Conventionally, the Potsic classification has been widely used in reports on CC. In a report by Wei et al. from China, the majority of cases were classified as stage III and IV of the Potsic classification [24]. It has been reported that CC is more prevalent in the anterior–superior quadrant (ASQ) of the tympanic cavity in Western countries. In contrast, it is more prevalent in the posterior–superior quadrant (PSQ) in Japan [8]. In addition, Hidaka et al. obtained similar results in a systematic review and meta-analysis comparing East Asia (Japan and Korea combined) and Western countries [23]. As in both Asian countries, in China, the primary prevalence of CC may also tend to be in PSQ. In our study, of the 47 JOS stage I ears, 18 ears were Ia (involved only in ASQ), 21 ears were Ib (involved only in PSQ), and 8 ears were Ic (involved in both ASQ and PSQ). In Hidaka’s own study in Japan, of all Potsic’s classified cases, 8 ears were involved only in ASQ, 12 ears were involved in PSQ, and 11 ears were involved in both ASQ and PSQ; consequently, the proportions of ASQ and PSQ were similar in both Japanese studies. Therefore, using the Potsic classification, Asian countries with a high prevalence of PSQ are more likely to have ossicular destruction and be classified as having an advanced stage. In 2017, a new classification and staging criteria were published by EAONO/JOS: stage I, cholesteatoma localized in the tympanic cavity; stage II, cholesteatoma involving two or more sites; stage III, cholesteatoma with extracranial complications; and stage IV, cholesteatoma with intracranial complications [6]. As this classification becomes more widely used in the future, the differences between countries may decrease.

A limitation of this study is that some patients may not have been included; for example, patients who moved out before being diagnosed (since this study was conducted in a rural area, there may be more people moving out than moving in), patients who were referred to hospitals in other larger cities, and patients whose disease progressed undiagnosed and could no longer be distinguished from acquired cholesteatoma. Koltai et al. reported two cases in which the lesion had significantly extended beyond the tympanic membrane and into the external auditory canal [9]. In addition, there have been reports of cases in which CC disappeared spontaneously [25]; therefore, there is a possibility that latent patients may be cured without being diagnosed or treated. In any case, the calculated incidence of CC may be considered lower than the actual number, as the number of cases may be underestimated but not overestimated. Thus, the incidence of CC calculated in this study can be considered a “minimum” value.

The method used in this study is not difficult. It would be easier to replicate the study in rural areas where patients with CC are concentrated in one large hospital. We list two items as directions for future research. First, conducting the same research in other countries will reveal any racial differences in the incidence of CC. Second, conducting the same research in later years may reveal the “true” incidence that has reached the upper limit.

## 5. Conclusions

We researched the epidemiology of CC based on the demographic trends across two prefectures in Japan. This kind of research could be carried out more advantageously in rural areas’ “closed” environments. The total incidence of CC was 26.44 per 100,000 newborns. No significant difference existed between the incidences in each prefecture, ensuring the reliability of the results. The incidence of CC tended to increase; however, more precisely, it can be considered an increase in the detection rate. Therefore, this number is expected to reach a plateau in the near future. The number of surgeries performed also increased significantly in the latter half of the study period; however, there was no significant change in patients’ ages at surgery. The most common first opportunities for medical examinations differed between before and after entering elementary school; the former were otitis media symptoms and routine examinations, while the latter was hearing loss. Hearing loss accounted for most opportunities for medical examinations after entering elementary school. We considered that this change was due to children becoming aware of hearing loss and screening conducted at schools. In this study, according to the JOS classification criteria, 47 ears were stage I, 53 ears were stage II, and there were not any stage III or IV cases. Unlike other studies using the Potsic classification, there was no relevance to age or progress.

## Figures and Tables

**Table 1:** Number of patients with CC, newborn births, and incidence.

Year of Birth	Patients in Oita	Newborn in Oita	Incidence in Oita	Patients in Miyazaki	Newborn in Miyazaki	Incidence in Miyazaki	Total Patients	Total Incidence
2001	3	10,891	27.55	0	11,007	0.00	3	13.7
2002	1	10,424	9.59	3	10,657	28.15	4	18.97
2003	3	10,213	29.37	1	10,220	9.78	4	19.58
2004	2	10,024	19.95	2	10,267	19.48	4	19.71
2005	0	9780	0.00	3	9738	30.81	3	15.37
2006	4	10,156	39.39	4	10,094	39.63	8	39.51
2007	2	10,162	19.86	6	10,337	58.04	8	39.03
2008	1	10,306	9.70	4	10,292	38.87	5	24.27
2009	1	9961	10.04	4	10,170	39.33	5	24.84
2010	2	10,072	19.86	2	10,217	19.58	4	19.72
2011	3	9988	30.04	1	10,152	9.85	4	19.86
2012	4	9650	41.45	1	9858	10.14	5	25.63
2013	4	9605	41.64	1	9854	10.15	5	25.70
2014	5	9279	53.89	5	9509	52.58	10	53.23
2015	3	9112	32.92	2	9226	21.68	5	27.27
2016	6	9059	66.23	3	8929	33.60	9	50.03
2017	3	8658	34.65	0	8797	0.00	3	17.19
Total	47	167,340	28.09	42	169,324	24.80	89	26.44

## Data Availability

The data presented in this study are available on request from the corresponding author.
